# Effect of antitumor therapy on cancer patients infected by SARS‐CoV‐2: A systematic review and meta‐analysis

**DOI:** 10.1002/cam4.3754

**Published:** 2021-02-06

**Authors:** Piao Li, Lingling Li, Shennan Wang, Yu Liu, Zhou Li, Shu Xia

**Affiliations:** ^1^ Department of Oncology Tongji Hospital Tongji Medical College of Huazhong University of Science and Technology Wuhan China; ^2^ Xiangyang Central Hospital Xiangyang China

**Keywords:** antitumor therapy, cancer, chemotherapy, COVID‐19, meta‐analysis, systematic review

## Abstract

**Background:**

Cancer patients are at a high risk of being infected with severe acute respiratory syndrome coronavirus 2 (SARS‐CoV‐2), and are more likely to develop severe illness and have higher mortality once infected. In the COVID‐19 pandemic, it is urgent to understand the effects of antitumor therapy on the prognosis of patients with COVID‐19.

**Methods:**

A systematic literature search was conducted in PubMed, Cochrane Library, Embase, MedRxiv, and Chinese National Knowledge Infrastructure (CNKI) until 21 June 2020. Odds ratios (ORs) and 95% confidence intervals (95% CIs) were evaluated using a random effects model to analyze the effects of antitumor therapies on COVID‐19 patients.

**Results:**

For cancer patients with COVID‐19, the death events related to antitumor treatment were higher than those with no antitumor treatment (OR = 1.55; 95% CI 1.07–2.25; *p* = 0.021). Compared with patients in the survival group, the non‐survival group showed no significant differences in patients who received antitumor therapy. Compared with patients in the non‐severe group, the severe group was more likely to receive antitumor therapy (OR = 1.50; 95% CI 1.02–2.19; *p* = 0.037) and there was a significant difference. The incidence of severe events was higher in the subgroup of chemotherapy (OR = 1.73; 95% CI 1.09–2.73).

**Conclusion:**

The synthesized evidence suggests that cancer patients with COVID‐19 who received antitumor treatment shortly before symptom onset are more likely to experience severe symptoms and have high mortality. Receiving chemotherapy is an unfavorable factor for the prognosis of cancer patients with COVID‐19.

## Graphical abstract

Patients with cancer and COVID‐19 who received anti‐tumor treatment shortly before symptom onset are more likely to develop severe illness and have high a mortality. Receiving chemotherapy is an unfavorable factor for the prognosis of patients with COVID‐19.

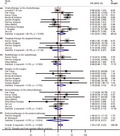

## 1 INTRODUCTION

In December 2019, a highly infectious beta‐coronavirus which causes the novel coronavirus disease 2019 (COVID‐19) was identified and named as severe acute respiratory syndrome coronavirus 2 (SARS‐CoV‐2).^1^, ^2^, ^3^ Almost the entire population lacks specific immunity to SARS‐CoV‐2 and can be infected by inhaling droplets containing coronavirus or by touching contaminated surfaces.^4^, ^5^ Subsequently, COVID‐19 spread rapidly across the world over the following months and was then recognized as a pandemic.^6^ As of 16 August 2020, there were 21,294,845 confirmed cases including 761,779 deaths, involving 216 countries.^7^

During the COVID‐19 pandemic, cancer patients still need to visit the oncology department for the timely diagnosis and treatment to avoid deterioration of their condition, which dramatically increases their risk of potential exposure to SARS‐CoV‐2. Multiple studies have shown that comorbidities are important risk factors for the morbidity and mortality of COVID‐19, among which the most challenging one may be immunosuppression.^8^, ^9^, ^10^, ^11^ Most patients with cancer tend to be old, and often suffer from immune dysfunction incurred by cancer or anticancer therapies.^12^ Therefore, the management strategy of cancer patients is incredibly essential in the COVID‐19 pandemic. A retrospective study by Liang et al. first pointed out that the incidence of COVID‐19 in cancer patients was higher than that in the general population.^13^ Subsequently, multiple studies with larger sample sizes have indicated that COVID‐19 patients with cancer are more likely to require mechanical ventilation, be admitted to the intensive care unit (ICU), and die.^14^, ^15^, ^16^, ^17^ However, further in‐depth investigations on the relationship between the antitumor treatment and the prognosis of COVID‐19 are limited and the conclusions are controversial. Our study aims to systematically review the present evidence on the relationship between antitumor therapy and the clinical outcomes of COVID‐19 and to analyze and summarize the available studies in this meta‐analysis.

## 2 METHODS

### 2.1 Literature search and study eligibility

This meta‐analysis was conducted according to the Preferred Reporting Items for Systematic Reviews and Meta‐Analyses (PRISMA) guidelines. We explored the literature database for studies published from 1 January 2020 to 21 June 2020, without language restrictions. The following search terms (“COVID‐19” OR “SARA‐CoV‐2” OR “severe acute respiratory syndrome coronavirus 2” OR “2019 novel coronavirus” OR “2019‐nCoV” OR “novel coronavirus”) AND (“Tumor” OR “cancer” OR “malignancy” OR “neoplasm”) were used in the databases including PubMed, Embase, Cochrane Library, Chinese National Knowledge Infrastructure (CNKI), and MedRxiv. We also examined the reference lists of related articles that described cancer patients with COVID‐19. The selection criteria for inclusion in this meta‐analysis were as follows: (a) the reported results included the death events of cancer patients who received antitumor treatment before diagnosis of COVID‐19; (b) the patients could be divided into severe and non‐severe groups, or survivors and non‐survivors groups; (c) the total number of patients included exceeded 10; and (d) the diagnosis of COVID‐19 was through an RT‐PCR test from a nose or throat swab and not from a radiological or clinical diagnosis. In the included articles, the severe group comprised patients with severe or critical COVID‐19, and the non‐severe group formed patients with mild or moderate COVID‐19. Two researchers independently completed the screening process by reviewing the abstracts and the full text of the articles. If there was a disagreement, a third researcher was consulted, and the controversy was resolved by consensus.

### 2.2 Information extraction and quality assessment

The following information was extracted: first author, publication year, country, number of patients, treatment, median age, percentage of males, and smoking status as current or former. We used the Newcastle–Ottawa Scale (NOS) to evaluate the quality of all eligible articles. It included the three aspects of selection, comparability, and exposure, with a full score of 9 points.

### 2.3 Statistical analysis

The odds ratios (ORs) and the 95% confidence intervals (CIs) were used as indicators. We used a random‐effects model to analyze antitumor therapy in patients with cancer and COVID‐19 in this meta‐analysis. Statistical heterogeneity was evaluated using *I^2^* and *P*‐values. *p* < 0.05 was considered statistically significant. Sensitivity analysis was performed to assess the reliability of the results. All statistical analyses were conducted using Stata statistical software (version 12.0; StataCorp LP).

## 3 RESULTS

### 3.1 Study eligibility and study characteristics

**FIGURE 1:**
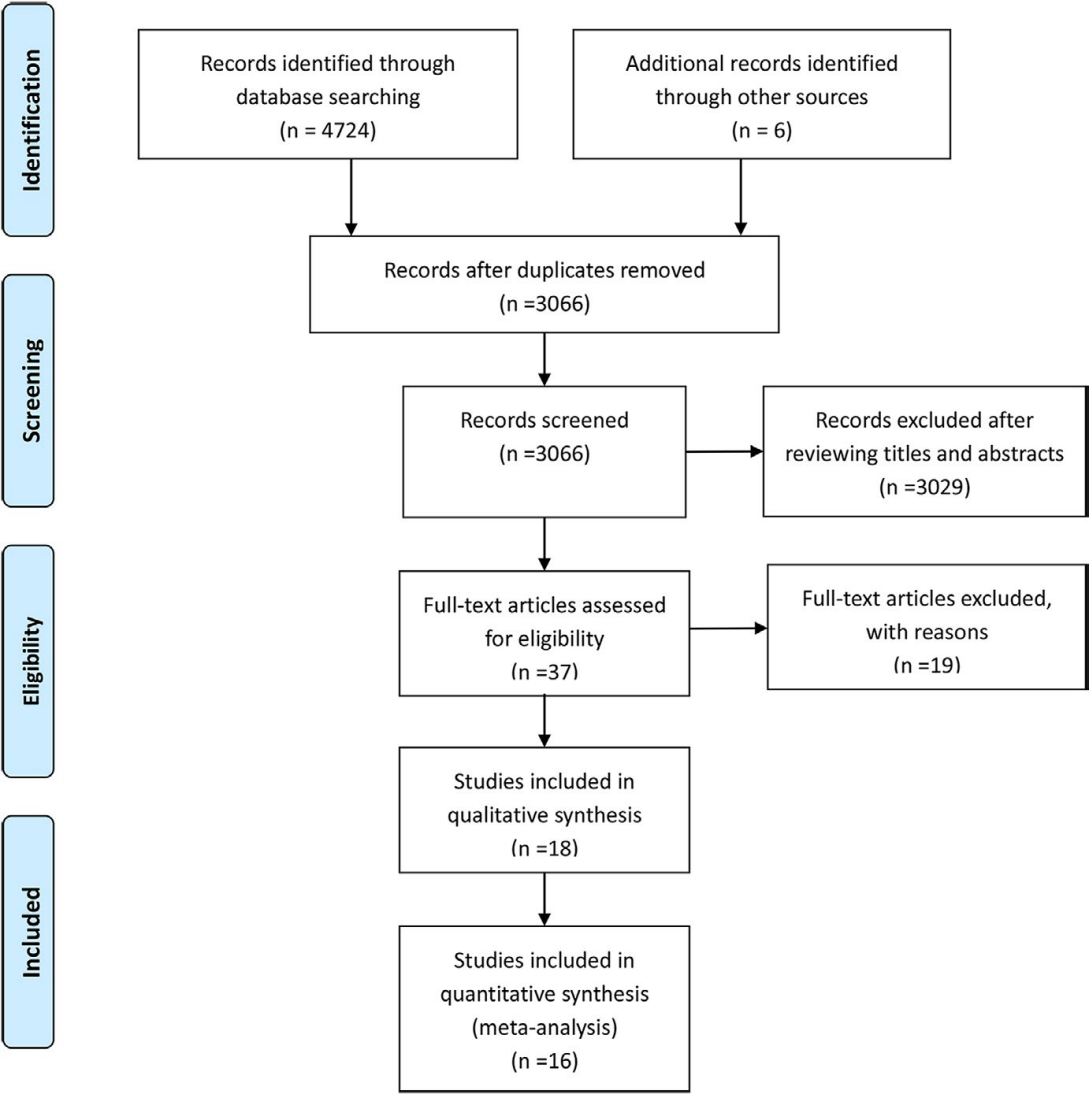
Flow chart of identified and included studies

Based on the search terms, a total of 4730 records were identified by searching the databases and references. A total of 3066 records were selected after removing the duplicate studies. Thirty‐seven articles were assessed based on their full text. Finally, 16 studies were included in this meta‐analysis (Figure 1). The primary characteristics of the included studies are shown in Table 1. A total of 3150 patients were included in this meta‐analysis. There were four studies from China; three from United States; two from United Kingdom; three from Italy; two from Spain; one from France; and one from United States, Canada, and Spain. The antitumor treatments were as follows: chemotherapy, targeted therapy, radiotherapy, surgery, immunotherapy, and endocrine therapy. All studies were considered high‐quality, and the NOS scores were more significant than 6 points (Table 2).

**TABLE 1:** Summary of all the basic characteristics of the included studies

First author	Year	Country	Number of patients	Treatment	Median age	Male (%)	Current or former smoking (%)
M. Montopoli	2020	Italy	118	Endocrinotherapy	73	100	NR
Lennard Y W Lee	2020	UK	800	Chemotherapy	69	56	NR
Mengyuan Dai	2020	China	105	Chemotherapy Targeted therapy Radiotherapy Surgery Immunotherapy Endocrinotherapy	61	56	33
Jia Luo	2020	USA	69	Chemotherapy Targeted therapy Immunotherapy	69	48	64
Fan Yang	2020	China	52	Chemotherapy Surgery Immunotherapy	63	53.8	NR
Jianbo Tian	2020	China	232	Chemotherapy Targeted therapy Radiotherapy Surgery Immunotherapy	64	51	6
Kunyu Yang	2020	China	205	Chemotherapy Targeted therapy Radiotherapy Surgery Immunotherapy	63	47	NR
Nicole M Kuderer	2020	USA Canada Spain	928	Chemotherapy Radiotherapy Surgery	66	50	40
Jacobo Rogado	2020	Spain	17	Chemotherapy Targeted therapy Surgery Immunotherapy	68	76.5	NR
Perrine Vuagnat	2020	France	59	Chemotherapy Targeted therapy Radiotherapy Surgery Endocrinotherapy	58	NR	NR
Vikas Mehta	2020	USA	218	Chemotherapy Radiotherapy Immunotherapy	69	58	NR
Elisa Maria Stroppa	2020	Italy	25	Chemotherapy Immunotherapy	72	80	52
J Rogado	2020	Spain	45	Chemotherapy	71	66.7	NR
Giorgio Bogani	2020	Italy	19	Chemotherapy Surgery	65	NR	NR
Jia Luo	2020	USA	102	Chemotherapy Targeted therapy Immunotherapy	68	48	27
B Russell	2020	UK	156	Chemotherapy Targeted therapy Immunotherapy	67	58	32

**TABLE 2:** Newcastle–Ottawa Scale (NOS) score of all eligible articles

Included study	Selection				Comparability	Exposure/outcome			Total scores
	Is the case definition adequate?/ascertainment of exposure	Representativeness of the cases/exposed cohort	Selection of controls/the non‐exposed cohort	Definition of controls/demonstration that outcome of interest was not present at start of study	Comparability of both groups/cohorts on the basis of the design or analysis	Ascertainment of exposure/assessment of outcome	same method of ascertainment for both groups/was follow‐up long enough for outcomes to occur	Non‐response rate/adequacy of follow up of cohorts	
M. Montopoli	*	*	*	*	—	*	*	—	6
Lennard Y W Lee	*	*	*	*	*	*	*	*	8
Mengyuan Dai	*	*	*	*	*	*	*	*	8
Jia Luo	*	*	*	*	*	*	*	*	8
Fan Yang	*	*	—	*	—	*	*	*	6
Jianbo Tian	*	*	*	*	**	*	*	*	9
Kunyu Yang	*	*	*	—	—	*	*	*	6
Nicole M Kuderer	*	*	*	*	**	*	*	*	9
Jacobo Rogado	*	*	*	*	—	—	*	*	6
Perrine Vuagnat	*	*	*	—	—	*	*	*	6
Vikas Mehta	*	*	*	*	*	—	*	*	7
Elisa Maria Stroppa	*	*	*	*	**	*	*	*	9
J. Rogado	*	*	*	*	—	—	*	*	6
Giorgio Bogani	*	*	*	*	—	*	*	—	6
Jia Luo	—	*	*	*	*	*	*	*	7
Russell B	*	*	*	*	—	*	*	*	7

### 3.2 Antitumor treatment‐related outcomes

The following treatment‐related outcomes were analyzed: the comparison of the death events between antitumor treatment and no antitumor treatment groups, the comparison between the non‐survival and survival groups with antitumor treatment, and the comparison between the severe and non‐severe groups with antitumor therapy in cancer patients with COVID‐19. Different treatment options were also conducted for the subgroup analyses.

### 3.3 Antitumor treatment and survivors and non‐survivors of COVID‐19

**FIGURE 2:**
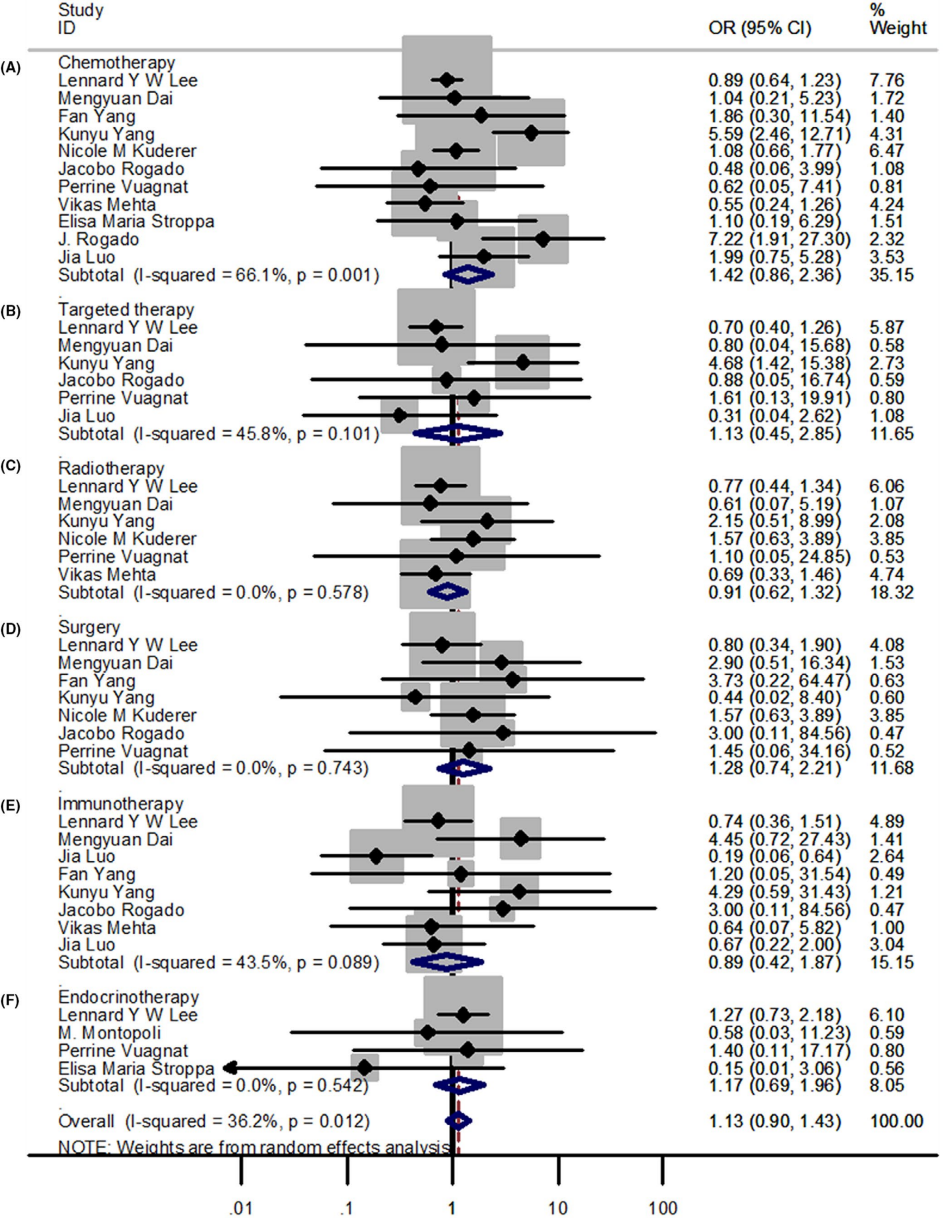
Forest plots of different treatments. (A‐F) Forest plot between non‐survivor and survivor groups for patients receiving chemotherapy, targeted therapy, radiotherapy, surgery, immunotherapy, and endocrine therapy; OR, Odds ratio

For patients with cancer and COVID‐19, there were no significant differences between the non‐survival and survival groups with chemotherapy (OR = 1.42; 95% CI 0.86–2.36), targeted therapy (OR = 1.13; 95% CI 0.45–2.85), radiotherapy (OR = 0.91; 95% CI 0.62–1.32), surgery (OR = 1.28; 95% CI 0.74–2.21), immunotherapy (OR = 0.89; 95% CI 0.42–1.87), and endocrine therapy (OR = 1.17; 95% CI 0.69–1.96) (Figure 2). Similarly, with antitumor treatment, the survival and non‐survival groups showed no significant differences (OR = 1.13; 95% CI 0.90–1.43; *p* = 0.294) in patients with cancer and COVID‐19.

### 3.4 Antitumor treatment and severe and non‐severe COVID‐19

**FIGURE 3:**
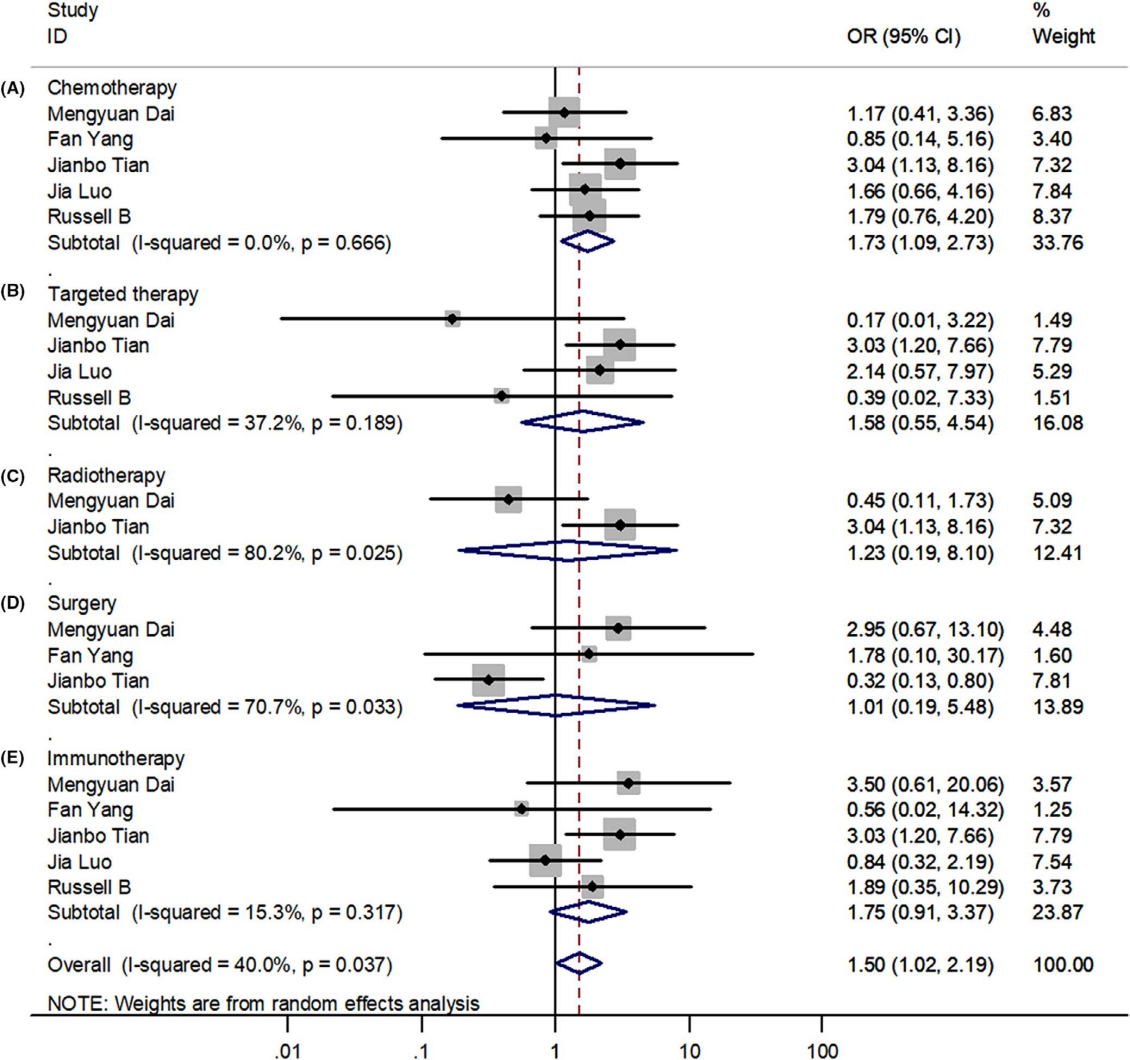
Forest plots of different antitumor therapies. (A‐E) Forest plot between severe and non‐severe groups for patients receiving chemotherapy, targeted therapy, radiotherapy, surgery, and immunotherapy; OR, Odds ratio

For the patients with cancer and COVID‐19, compared with the non‐severe group, the incidence of severe events was higher in the subgroup of chemotherapy (OR = 1.73; 95% CI 1.09–2.73) (Figure 3). However, there was no significant difference between the severe and non‐severe groups with targeted therapy (OR = 1.58; 95% CI 0.55–4.54), radiotherapy (OR = 1.23; 95% CI 0.19–8.10), surgery (OR = 1.01; 95% CI 0.19–5.48), and immunotherapy (OR = 1.75; 95% CI 0.91–3.37). The analysis of endocrine therapy was not performed due to insufficient data. However, for antitumor treatment, compared with patients in the non‐severe group, the severe group had a higher incidence (OR = 1.50; 95% CI 1.02–2.19; *p* = 0.037) with significant differences.

### 3.5 Death events between antitumor and non‐antitumor therapies

**FIGURE 4:**
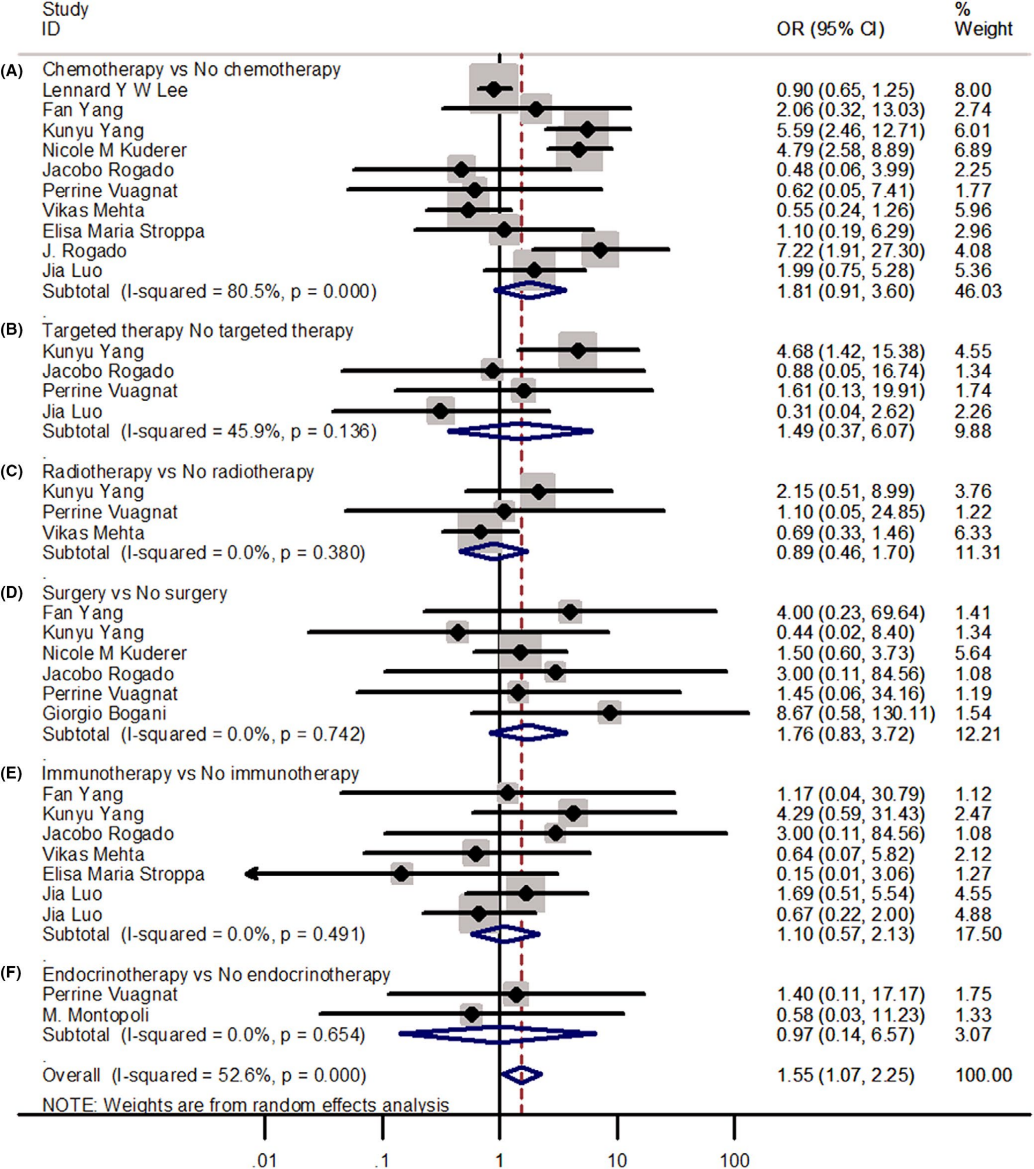
Forest plots of odd ratios for death events between patients receiving and not receiving antitumor therapy. (A‐F) Forest plot of analyses of the death events between patients receiving and not receiving chemotherapy, targeted therapy, radiotherapy, surgery, immunotherapy, and endocrine therapy; OR, Odds ratio

The number of death events in patients receiving chemotherapy was higher than that in patients without chemotherapy; however, the result was not statistically significant (OR = 1.81; 95% CI 0.91–3.60) (Figure 4). Meanwhile, compared with the non‐antitumor treatment group, the antitumor treatment group demonstrated no significant difference in death events with targeted therapy (OR = 1.49; 95% CI 0.37–6.07), radiotherapy (OR = 0.89; 95% CI 0.46–1.70), surgery (OR = 1.76; 95% CI 0.83–3.72), immunotherapy (OR = 1.10; 95% CI 0.57–2.13), and endocrine therapy (OR = 0.97; 95% CI 0.14–6.57) in the patients with cancer and COVID‐19. However, the overall effect was that the mortality of antitumor treatments was higher than that of no antitumor treatment in the patients with COVID‐19, and the result was statistically significant (OR = 1.55; 95% CI 1.07–2.25; *p* = 0.021).

### 3.6 Sensitivity analysis

**FIGURE 5:**
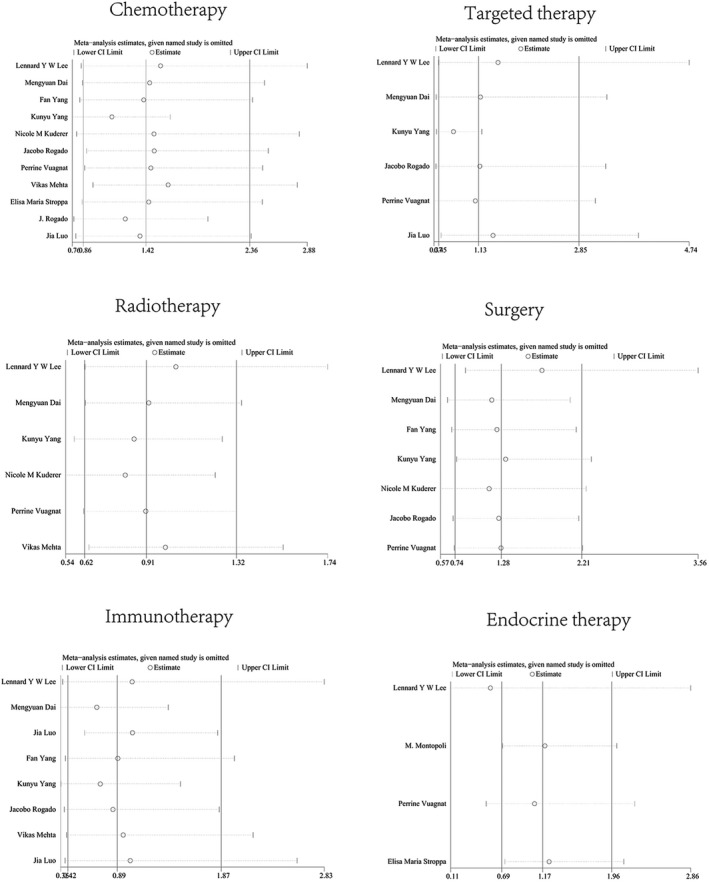
Results of the sensitivity analysis

The results of sensitivity analysis confirmed that the outcomes were not influenced by eliminating any one specific study in chemotherapy, targeted therapy, radiotherapy, surgery, immunotherapy, and endocrine therapy between non‐survival and survival groups (Figure 5).

## 4 DISCUSSION

To our knowledge, this is a relatively comprehensive meta‐analysis that explored the association between different antitumor treatments and the prognosis of COVID‐19. This meta‐analysis of 16 studies involving 3150 patients with cancer indicated that COVID‐19 patients on antitumor therapy were more likely to develop severe events and have an increased likelihood of death. We further analyzed the impact of different antitumor therapies on the prognosis of COVID‐19. Cancer patients with COVID‐19 who recently received chemotherapy were more likely to develop severe illnesses. The mortality of patients on chemotherapy was higher than that of the patients not on chemotherapy; however, the result was not statistically significant.

So far, the COVID‐19 pandemic is still ongoing and constitutes a public health emergency of international concern.^18^ Some countries that thought they had effectively curbed the spread of the virus are now experiencing a resurgence of the COVID‐19 epidemic. Some areas were less affected in the early stage of the COVID‐19 pandemic, but now the number of cases and deaths are rising sharply. Dr Tedros said that COVID‐19 is a once‐in‐a‐century crisis for humanity and its impact will last for decades.^19^ Although COVID‐19 biomedical research is currently being carried out urgently, till date, there are no vaccines and effective therapies. As the number of COVID‐19 cases and affected regions continue to increase, special preparations should be made to protect high‐risk groups, especially elderly patients and patients with underlying diseases.

The incidence and mortality of cancer are increasing rapidly worldwide. It has been found that cancer is one of the main risk factors for COVID‐19 patients.^13^, ^14^, ^20^, ^21^, ^22^ A retrospective study first indicated that cancer patients had a higher risk of SARS‐CoV‐2 infection than those without cancer and had worse outcomes once infected,^13^ while subsequent studies with larger sample sizes yielded the same results.^21^, ^23^ These data provide early insights into how COVID‐19 may affect the management of cancer patients. After correcting for publication bias and adjusting for heterogeneity, a meta‐analysis drew a conclusion that cancer survivors have a higher risk of COVID‐19, and are related to cases of severe and risk of death.^24^ It has been recommended to modify the method of administration and the intervals between antitumor treatment according to the condition of cancer patient to reduce the risk of contracting COVID‐19 through the hospital.^25^, ^26^

Based on the above findings, multiple studies have further explored the effects of antitumor therapies, in‐depth, on COVID‐19, yet the conclusions are controversial. Some studies had shown that there was no evidence of increased mortality rates among patients with COVID‐19 on anticancer treatments.^16^, ^25^, ^27^ A multicenter, retrospective cohort study showed that receiving chemotherapy within 28 days before symptom onset was correlated with increased mortality of COVID‐19 during admission to hospital.^17^ A meta‐analysis conducted by Venkatesulu et al. showed that cancer patients were at an increased risk of mortality and morbidity from COVID‐19, but there was no correlation between a specific type of antitumor treatment and mortality.^28^ Based on this study, our study included more recent evidence to analyze further the relationship between antitumor treatment and the prognosis of COVID‐19. Our investigation indicated that receiving antitumor treatment was associated with the severity of COVID‐19. Among cancer patients with COVID‐19 receiving antitumor treatment, there was a trend toward higher the non‐survival group with patients in survival group (OR = 1.13; 95% CI 0.90–1.43; *p* = 0.294). Notwithstanding, the result was not statistically significant. However, after dividing different antitumor treatment methods into subgroups for analysis, we found that only chemotherapy showed significant associations with the incidence of severe illness. We also observed a numerical increase in mortality among patients who recently received chemotherapy, and the result was not statistically significant. Consequently, it is necessary to establish large‐scale clinical trials to provide more reliable evidence for the choice of antitumor treatment for cancer patients with COVID‐19. The immunity of patients with COVID‐19 may be a key factor for their prognosis. Clinically, in non‐severe stages of COVID‐19, the adaptive immune response is essential to clear the SARS‐CoV‐2 and prevent patients from deteriorating.^29^ At this stage, a healthy host with a useful immune function can eliminate the virus by developing an endogenous protective immune response. Patients undergoing chemotherapy often suffer from bone marrow suppression and impaired immunity.^30^ After being infected with SRS‐CoV‐2, they cannot effectively activate the immune system to eliminate the virus, so they are more likely to experience severe symptoms or even death.

In our study, cancer patients diagnosed with COVID‐19 who received immunotherapy tended to have a higher rate of severe illness than those who did not receive immunotherapy; however, there was no significant difference. In the past decade, tumor immunotherapy has developed rapidly and become an important antitumor treatment method.^31^ During the COVID‐19 pandemic, the use of immune checkpoint inhibitors is a controversial issue, as they are a class of molecules that directly target and regulate the immune response. Immunotherapy can reactivate immunity, in particular, T cell‐mediated immunity, which helps to resist viruses.^32^, ^33^, ^34^ Based on this view, immunotherapy may benefit patients with COVID‐19.^35^ On the other hand, theoretically, immunotherapy could induce excessive activation of the immune system during COVID‐19 (i.e., the cytokine storm), which may worsen the outcome of the COVID‐19 infection.^36^ Furthermore, immunotherapy may induce immune‐related adverse events, including interstitial pneumonitis, which may have a negative synergistic effect on a SARS‐CoV‐2 infection.^37^ Clinically, the immune response of COVID‐19 is mainly divided into two stages.^29^ In the non‐severe stage, useful immune function helps patients against the virus and prevent deterioration of the condition. However, during the severe stage, proper immune function may aggravate lung inflammation. Therefore, immunotherapy may have different effects at different stages of COVID‐19. While further studying the impact of immunotherapy on COVID‐19, it is recommended to conduct more intensive monitoring for patients receiving immunotherapy during the COVID‐19 pandemic.

Our data suggested that targeted therapy, radiotherapy, and surgery did not affect the clinical outcomes of cancer patients with COVID‐19. However, it is worth noting that some included studies lack information of different antitumor treatments, so it is impossible to clarify its relationship with COVID‐19. More detailed data with larger numbers of COVID‐19 patients with cancer are needed for an accurate verdict. It is vital to carefully reduce the number of hospital visits for cancer patients to protect them from exposure to SARS‐CoV‐2. For patients with COVID‐19 who recently received antitumor treatment, especially chemotherapy, clinicians should pay attention to disease progression and should focus on timely intervention. Stopping effective antitumor therapy for cancer patients during the pandemic may increase the risk of cancer‐related mortality, which may be far greater than COVID‐19 itself. Clinicians should carefully decide on suspending or extending the antitumor regimens of patients with cancer.

Large‐scale data from multiple centers are needed to clarify the relationship between tumor, antitumor therapies, and COVID‐19. Future studies should investigate the heterogeneity between different cancer types in COVID‐19 infectors, elucidate the impact of specific antitumor therapy on COVID‐19, discover whether scheduling of antitumor treatment affects the prognosis of COVID‐19, and better understand the interaction between host immune response and COVID‐19 in cancer patients.

### 4.1 Limitation

Our study has certain limitations. Firstly, the sample size of the included studies was relatively small, and much of the data were derived from subgroup analyses, which led to certain biases in the conclusions. Secondly, most of the included studies were single‐center studies with high selective reporting of data and publication bias. Thirdly, there was a lack of detailed information on antitumor treatments for different tumor types in the included studies. Due to limited data, we had not conducted subgroup analysis of antitumor treatments in different cancer types. Fourthly, there was also no information about death from COVID‐19 or cancer progression and cancer treatment‐related complications. We did not classify the causes of death. Meanwhile, the incidence and mortality of patients with advanced cancer might affect the severity and mortality of COVID‐19. Finally, most of the studies included in this meta‐analysis were retrospective studies, which weakened the reliability of the conclusions. However, the sensitivity analysis showed that the results of this meta‐analysis were reliable.

## 5 CONCLUSION

Our study indicated that antitumor treatment is associated with disease severity and risk of death of cancer patients with COVID‐19 infection. The odds of progressing to severe disease in cancer patients with COVID‐19 infection who recently received chemotherapy were higher than those of patients who did not receive chemotherapy. Our research aims to explore the effect of cancer treatments on the clinical outcomes of COVID‐19, then to assist clinicians in monitoring and evaluating the prognosis of patients with cancer and COVID‐19, and to guide clinicians to make the best decisions.

## CONFLICT OF INTEREST

The authors made no disclosures. All analyses were based on previous published studies, thus no ethical approval and patient consent are required.

## Data Availability

All data were included in the manuscript and there was no restriction for availability.
